# Markov Model Predicts Changes in STH Prevalence during Control Activities Even with a Reduced Amount of Baseline Information

**DOI:** 10.1371/journal.pntd.0004371

**Published:** 2016-04-01

**Authors:** Antonio Montresor, Arminder Deol, Natacha à Porta, Nam Lethanh, Dina Jankovic

**Affiliations:** 1 Department of Control of Neglected Tropical Diseases, World Health Organization, Geneva, Switzerland; 2 Schistosomiasis Control Initiative, Department of Infectious Disease Epidemiology, School of Public Health, Imperial College London, London, United Kingdom; 3 Swiss Federal Institute of Technology, Zurich, Switzerland; 4 Centre for Health Economics, University of York, York, United Kingdom; University of Warwick, UNITED KINGDOM

## Abstract

**Background:**

Estimating the reduction in levels of infection during implementation of soil-transmitted helminth (STH) control programmes is important to measure their performance and to plan interventions. Markov modelling techniques have been used with some success to predict changes in STH prevalence following treatment in Viet Nam. The model is stationary and to date, the prediction has been obtained by calculating the transition probabilities between the different classes of intensity following the first year of drug distribution and assuming that these remain constant in subsequent years. However, to run this model longitudinal parasitological data (including intensity of infection) are required for two consecutive years from at least 200 individuals. Since this amount of data is not often available from STH control programmes, the possible application of the model in control programme is limited. The present study aimed to address this issue by adapting the existing Markov model to allow its application when a more limited amount of data is available and to test the predictive capacities of these simplified models.

**Method:**

We analysed data from field studies conducted with different combination of three parameters: (i) the frequency of drug administration; (ii) the drug distributed; and (iii) the target treatment population (entire population or school-aged children only). This analysis allowed us to define 10 sets of standard transition probabilities to be used to predict prevalence changes when only baseline data are available (simplified model 1). We also formulated three equations (one for each STH parasite) to calculate the predicted prevalence of the different classes of intensity from the total prevalence. These equations allowed us to design a simplified model (SM2) to obtain predictions when the classes of intensity at baseline were not known. To evaluate the performance of the simplified models, we collected data from the scientific literature on changes in STH prevalence during the implementation of 26 control programmes in 16 countries. Using the baseline data observed, we applied the simplified models and predicted the onward prevalence of STH infection at each time-point for which programme data were available. We then compared the output from the model with the observed data from the programme.

**Results:**

The comparison between the model-predicted prevalence and the observed values demonstrated a good accuracy of the predictions. In 77% of cases the original model predicted a prevalence within five absolute percentage points from the observed figure, for the simplified model one in 69% of cases and for the simplified model two in 60% of cases. We consider that the STH Markov model described here could be an important tool for programme managers to monitor the progress of their control programmes and to select the appropriate intervention. We also developed, and made freely available online, a software tool to enable the use of the STH Markov model by personnel with limited knowledge of mathematical models.

## Author Summary

Several million children are periodically dewormed to prevent the impairments to health and the economy caused by soil-transmitted helminths in endemic communities. It is important that managers of STH control programmes be able to anticipate the impact of the control measures on the prevalence of the diseases for two main purposes:

- to select the most appropriate intervention; and
- to be alerted in case of changes in prevalence that are less favourable than the expected values.

We therefore developed a model that predicts such changes, and also included modifications to enable its use in cases where a limited amount of data is available (SM1 and SM2). We retrospectively tested the performances of the model on 26 control programmes comparing the prevalence predicted by the model with those observed during programme implementation: in 77% of cases the prevalence predicted by the original model was within five percentage points of the observed values (69% in SM1 and 60% in SM2). We consider the performances of these models to be satisfactory. We designed a free online software to facilitate the use of these models by programme managers who may not be confident with modelling procedures.

## Introduction

Soil-transmitted helminths (STHs) is a group of four species of intestinal worms: *Ascaris lumbricoides*, *Trichuris trichiura*, *Necator americanus* and *Ancylostoma duodenale*. The latter two species are indistinguishable by microscopic examination of the eggs and are therefore frequently reported collectively as “hookworms”. STH infections cause morbidity by adversely affecting nutritional status and impairing cognitive processes [1].

The World Health Organization (WHO) recommends preventive chemotherapy (that is, the periodic administration of anthelminthic medicines) as a public health intervention for the control of STH [2]. The aim of the intervention is to reduce the number of individuals harbouring infections of moderate and heavy intensity and therefore to control the morbidity caused by STH infection [3].

We previously evaluated the performance of a Markov model in predicting the changes in STH prevalence during the implementation of preventive chemotherapy in Viet Nam over 54 months. The model is described fully elsewhere [4].

Predicting the short- to medium-term (1–5 years) changes in STH prevalence could facilitate the work of control programme managers in selecting the most appropriate intervention and in monitoring the ongoing outcomes of the programme.

In all the datasets used in the present study (i.e. those from the studies used to calculate the model parameters and those from control programmes used to evaluate the prediction capacity of the model), Kato-Katz was the only method used to collect parasitological data. We understand the poor precision of the diagnostic methods traditionally used to monitor STH control programmes (i.e. Kato-Katz) especially when intensities of infection are low [1].

We recognize that the prevalence would have been higher if measured with a more precise method than Kato-Katz, but we wanted to provide programme managers with a tool capable of interpreting the imperfect data collected during routine control activities. The model requires pre-control data on the distribution of intensity of infection in the population, defined as the proportion of the population with zero eggs, light intensity, moderate intensity and high intensity of infection as defined by WHO [2] for each of the three STH parasites (Table 1). The Markov nomenclature refers to these classes of intensity as “condition states” (CS).

**Table 1:** WHO classification of intensity of soil-transmitted helminth (STH) infections corresponding to condition states in the Markov nomenclature [1].

	Condition State 1	Condition State 2	Condition State 3	Condition State 4
STH species	Zero eggs (epg)	Infections of light intensity (epg)	Infections of moderate intensity (epg)	Infections of heavy intensity (epg)
*A*. *lumbricoides*	0	1–4999	5000–49 999	> 50 000
*T*. *trichiura*	0	1–999	1000–9999	> 10 000
Hookworms	0	1–1999	2000–3999	> 4000

epg = eggs per gram of faeces.

**Fig 1:**
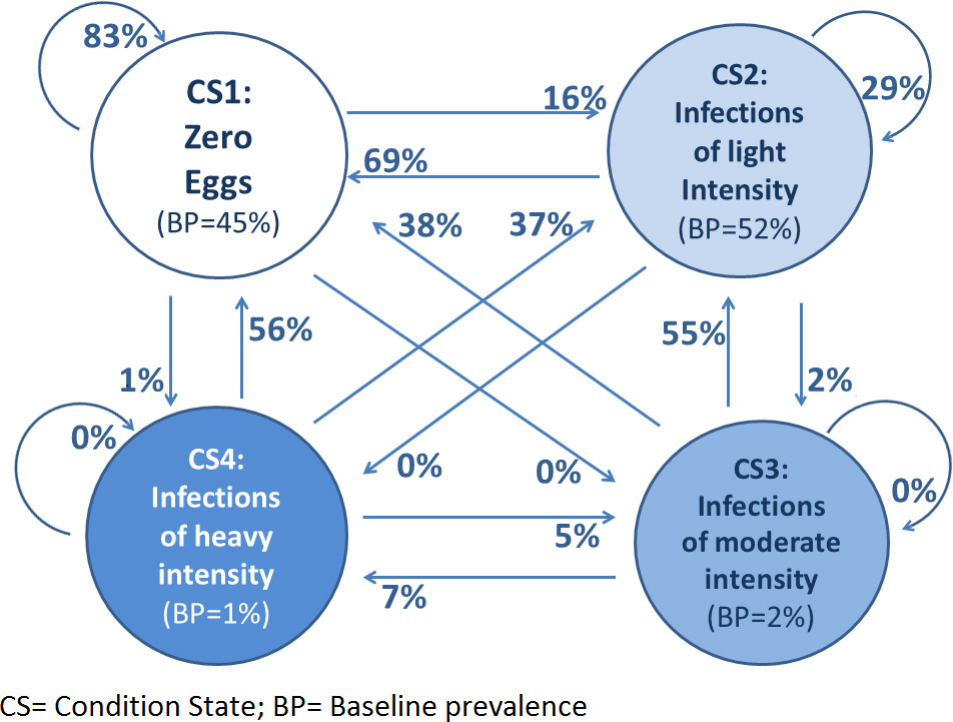
Condition states (represented by the four circles) and transition probabilities (represented by the 16 arrows) for cases of hookworm infection treated with albendazole once per year (data from Uganda). The CS and TP were calculated by analysing individual baseline and follow up data from 1423 individuals. The baseline prevalence of hookworm infection was 55% (52% of light-intensity infection, 2% of moderate-intensity infection and 1% of heavy-intensity infection). After one year of implementation of control measures, 69% of the light-intensity infections (CS2) were reduced to “zero eggs”, 29% of the CS2 remained as light-intensity infections and 2% increased to moderate-intensity infections.

Initially, each individual in a specified population is categorized into one of the four CS but may transition to another CS after the first year of intervention: the changes between these four CS are defined as transition probabilities (TP); the 16 TP corresponding to each of the possible changes are captured in a four-by-four TP matrix. Fig 1 illustrates the TP and the CS for hookworm infection in Uganda resulting from the distribution of albendazole once a year among 1423 school-aged children individually followed for 3 years [5].

A transition probability matrix set (TPMS) is composed of three TP matrices (one matrix for each STH species). The Markov model assumes the “stationarity” of the TP, in the sense that if the intervention remains the same (e.g. albendazole is administered once a year) the TPMS will also remain constant throughout the duration of the control programme. For example, Fig 1 shows that if 37% of heavy-intensity hookworm infections become light-intensity infections after the first year of preventive chemotherapy, the same proportion (37%) of the remaining heavy-intensity infections will become light-intensity infections after the second year of the intervention.

This assumption is based on:

1. the remarkable capacity of STH to contaminate the environment: a soil survey conducted in 2013 in the Philippines in an area with STH prevalence of 44% found STH eggs in 85% of soil samples collected inside houses, in backyards or from the paths around the village [6].
2. the long survival time of STH eggs in the environment: several weeks for hookworms [7], a few years for *T*. *trichiura* [8] and more than 10 years for *A*. *lumbricoides* [9].

Additionally, we assume that the pharmacological interventions conducted in only a part of the population (i.e. school-aged children) do not reduce significantly the faecal contamination of the soil in the short term.

In a previous study the Markov model with constant TPMS predicted changes of prevalence in Viet Nam at four time-points (6, 12, 30 and 54 months) for each STH infection with an average precision of 1.7 percentage points [4]. In this study we aimed to test the model’s capacity to predict changes in STH prevalence during implementation of control programmes in other countries.

One of the limitations of the model in its initial form is the requirement for a detailed dataset to calculate the TPMS: the minimum requirement to run the model is individual data (including the intensity of infection for each STH parasite) of at least 200 individuals, collected before and one year after the intervention.

However, the epidemiological data available in STH control programmes are often limited, which can thus limit the application of the model. We therefore adapted the Markov model to also allow predictions in cases when a more limited amount of data is available. For this purpose we developed two simplified models in addition to the model initially developed and now titled Original Model (OM):

- Simplified Model 1 (SM1) allows for the estimation of changes in STH epidemiology when baseline infection data (including classes of intensity of infection) are available but the epidemiological status after one year of intervention is not known. In other words when it is possible to calculate the baseline CS but not the TP.
- Simplified Model 2 (SM2): allows estimation of changes in STH epidemiology when baseline prevalence data are available but not classes of intensity (CS) at baseline or of changes occurring after one year of intervention. In other words when it is not possible to calculate the baseline CS and TP from the dataset.

Table 2 summarizes the characteristics of the three models.

**Table 2:** Minimum requirements to run original model (OM), simplified model 1 (SM1) and simplified model 2 (SM2).

Model	Minimum requirements	
	Baseline data	1 year follow-up data
OM	Survey of 200 individuals, for each individual: intensity of *A*. *lumbricoides* infection, intensity of *T*. *trichiura* infection and intensity of hookworm infection	Survey of the same 200 individuals, for each individual: intensity of *A*. *lumbricoides* infection, intensity of *T*. *trichiura* infection and intensity of hookworm infection
SM1	Survey of 125 individuals, for each STH parasite: prevalence of light-intensity infection, prevalence of moderate-intensity infection and prevalence of high-intensity infection	No follow-up data required
SM2	Survey of 125 individuals, prevalence of *A*. *lumbricoides*, prevalence of *T*. *trichiura* and prevalence of hookworms	No follow-up data required

We intend the model to be a simple tool to be used by programme managers in developing countries, and we therefore designed a software tool to enable its use by personnel with limited familiarity with computer modelling.

## Material and Methods

### Original model (OM)

The OM was developed and tested on data from Viet Nam with satisfactory results. The model calculates the TP from individual data at baseline and one year after the intervention. The mathematical aspects of this model are presented in S1 Additional File.

### Simplified model (SM1)

The SM1 was developed to run when one-year follow up data are not available and the calculation of TP is therefore not possible from the programme data.

We developed 10 “standard” TPMS, one for each possible preventive chemotherapy intervention (Table 3) for use instead of TPMS from programme data.

**Table 3:** Standard transition probability matrix sets (TPMS) and the datasets used to develop TPMS.

TPMS	First round of drug distribution	Second round of drug distribution	Dataset used to develop standard TPMS	no. of school-aged children	Comments (assumption)	Ref.
TPMS 1	Albendazole	Albendazole	Viet Nam	366	Calculated form study implementing the intervention	[10]
TPMS 2	Mebendazole	Mebendazole	Tanzania (Zanzibar)	1311	Calculated form study implementing the intervention	[11]
TPMS 3	Albendazole	No second round intervention	Uganda	1423	Calculated form study implementing the intervention	[5]
TPMS 4	Mebendazole	No second round intervention	Tanzania (Zanzibar)	1324	Calculated form study implementing the intervention	[12]
TPMS 5	Albendazole + Ivermectin*	Albendazole	Viet Nam	366	Similar to TPMS1 (slightly more	[10–13]
			Tanzania (Zanzibar)	301	active in hookworms)	
TPMS 6	Albendazole + Ivermectin*	Mebendazole	Viet Nam and	366	Equal to TPMS5	[10–11]
			Tanzania (Zanzibar)	1311		
TPMS 7	Albendazole + Diethylcarbamazine*	Albendazole	Viet Nam	366	Equal to TPMS1 (absence of impact of DEC on STH)	[10]
TPMS 8	Albendazole + Diethylcarbamazine*	Mebendazole	Tanzania (Zanzibar)	1311	Equal to TPMS2 (absence of impact of DEC on STH)	[11]
TPMS 9	Albendazole + Ivermectin*	No second round intervention	Tanzania (Zanzibar)	301	Calculated form study implementing the intervention	[13]
TPMS 10	Albendazole+ Diethylcarbamazine*	No second round intervention	Uganda	1423	Equal to TPMS3 (absence of impact of DEC on STH)	[5]

* Interventions administered to the entire population in a district in the context of the global programme for elimination of lymphatic filariasis; their impact on STH prevalence in the community is larger than the administration of benzimidazoles to children only.

For four types of intervention the TPMS were directly measured from control programmes for which baseline date and one-year follow-up data were available:

- TPMS 1 (albendazole twice yearly) [10]
- TPMS 2 (mebendazole twice yearly) [11]
- TPMS 3 (albendazole once yearly) [5]
- TPMS 4 (mebendazole once yearly) [12]
- TPMS 9 (albendazole plus ivermectin first round; no treatment second round) [13]

For the other five types of intervention (TPMS 5,6,7,8 and 10) we could not measure the TP directly but we estimated the impact of the intervention applying two assumptions:

- When ivermectin with albendazole is provided during the first round of treatment and albendazole or mebendazole is provided during the second round of treatment the impact is similar to TPMS1/TPMS2 with a marginal additional efficacy against hookworm infection [13].
- The impact of diethylcarbamazine on STH is considered absent [1].

Table 3 summarizes the characteristics of the standard TPMS and the field study data used to develop them.

The mathematical aspects of this model are presented in S1 Additional File.

### Simplified Model 2 (SM2)

The SM2 was developed to run when baseline data relating to the intensity of infection are not available and the calculation of the CS is therefore not possible from the programme data.

The model also assumes that one-year follow up data are not available and uses the TPMS to run the SM1.

Equations predicting the relationship between baseline prevalence and CS at baseline were developed using data from 18 countries; details of the methodology are published elsewhere [14]. The mathematical aspects of this model are presented in S1 Additional File; S2 Additional File, presents the values of coefficients used for Simplified Model 1 (SM1) and Simplified Model 2 (SM2).

The two simplified models (SM1 and SM2) do not calculate specific TPMS for the control programme but apply standard TPMS that are calculated from programmes using a similar intervention; for example, to estimate the change in STH prevalence in Myanmar, the TPMS developed from the data from Viet Nam (TPMS1) were applied because the intervention in the two countries is the same (albendazole two rounds per year); to predict the change in prevalence in Cambodia the TPMS developed from the data from Zanzibar were applied (TPMS4).

### Coverage

Programme coverage (that is, the percentage of individuals treated in the target group) is an important parameter to be considered.

If, for example, only 50% of the school-aged population is treated, we would expect changes in the intensity of STH infection only in the individuals that have been treated.

For this purpose our model offers the functionality to use the coverage of the intervention to modify the output (the TP matrices would then be applied only to a fraction of the individuals corresponding to the coverage).

### Adaptation of the TPMS when the entire population is treated

During the development of the model we observed that when the entire population is treated (as is the case when albendazole is distributed for control of lymphatic filariasis), the predicted prevalence was consistently higher than the observed values; this discrepancy between the two predictions was increasing at the increase of the baseline prevalence. We interpret this discrepancy as a progressive decrease of reinfection due to the reduction of soil contamination consequent to administration of anthelminthics to the entire population. We adjusted for this factor by including a standard modification, dependent on the initial level of prevalence: if baseline prevalence is > 80% the standard modification becomes active after 5 years of follow up; for lower initial prevalence the standard modification becomes active after 3–4 years of follow up. This modification is only applied when the entire population is treated.

### Testing of the model

We hypothesize that the simplified models (SM1 and SM2), despite using standard TPMS, could predict with sufficient precision the changes in STH prevalence over 6 years.

To test the performances of the models we collected data from STH control programmes reporting the prevalence collected after a number of years of preventive chemotherapy from published and unpublished reports to extract the STH baseline epidemiology, the drug or drug combinations used, the type of administration (community or school-based) and the frequency of the intervention. These programme data are from countries different than the ones used to define the model parameters.

In total we obtained information from 45 follow-up surveys (with their respective baselines) in 14 countries [5, 15–31]: for three countries control programme managers provided the data [5, 15, 31]; for the remainder the datasets were obtained from published scientific literature [16–30]. Some baseline data corresponded to more than one follow-up: on average each baseline corresponded to 1.7 follow ups (range 1–5). The total number of individuals from whom data were collected was 36 108, with an average sample size of 508 for each survey (range 50–2885 individuals). Table 4 presents details of the datasets used to test the model. The datasets used to develop the standard TPMS (Table 3) or to estimate the CS [6] were not used for the evaluation of the model predictions.

**Table 4:** Datasets used to test the model.

Country	Sample size	No. of surveys		Baseline prevalence			Drug used	Number of years of intervention (years)	Frequency of intervention	Model tested	Ref.
		Baseline	Follow-up	*A. lumbricoides*	*T*. *trichiura*	Hookworms					
Viet Nam	210–261	1	5	30%	29%	76%	ALB	1–6	2/year	OM-SM1-SM2	[10, 15]
Uganda	1423	1	3	2%	2%	59%	ALB	1–3	1/year	OM-SM1-SM2	[5]
Zambia	490	1	2	22%	0	7.8%	ALB+IVR	1.5	1/year	SM1-SM2	[16]
Lao PDR	2885	1	1	61%	43%	19%	ALB	2	2/year	SM1-SM2	[17]
Myanmar, Pin Lung	250	1	1	23%	3%	5%	ALB	5	2/year	SM1-SM2	[18–19]
Myanmar Phhyu	250	1	1	50%	55%	10%	ALB	5	2/year	SM1-SM2	[18–19]
Myanmar, Nyangdone	250	1	1	63%	88%	0%	ALB	5	2/year	SM1-SM2	[18–19]
Myanmar, Meik	250	1	1	58%	84%	12%	ALB	5	2/year	SM1-SM2	[18–19]
UR Tanzania	1192	1	1	98%	90%	97%	ALB+IVR	13	1/year	SM1-SM2	[20]
Nepal	711	1	1	22%	19%	64%	ALB	2	1/year	SM1-SM2	[21]
India, A. Pradesh	217	1	1	73%	67%	0	ALB	0.75	1/year	SM1-SM2	[22]
India, W. Bengala	126	1	2	54%	0	0	ALB	0.5–1	2/year	SM2	[23]
India, Tamil Nadu	325	1	2	55%	17%	5%	ALB+DEC	1–2	1/year	SM2	[24]
Cambodia, Achen	50	1	1	25%	2%	55%	MEB	5	1/year	SM2	[25]
Cambodia, Chartnol	50	1	1	10%	2%	46%	MEB	5	1/year	SM2	[25]
Cambodia, Sambock	50	1	1	16%	2%	45%	MEB	5	1/year	SM2	[25]
Cambodia, Srekhoeun	50	1	1	14%	2%	50%	MEB	5	1/year	SM2	[25]
Cambodia, Sdau	50	1	1	51%	2%	86%	MEB	5	1/year	SM2	[25]
Cambodia, Koh Sneg	50	1	1	34%	2%	55%	MEB	5	1/year	SM2	[25]
Cambodia, K. Chan Tuk	50	1	1	70%	2%	67%	MEB	5	1/year	SM2	[25]
Seychelles, children	1058	1	2	18%	53%	6%	MEB	5	2/year	SM2	[26]
Seychelles,women	338	1	1	10%	36%	9%	MEB	2	2/year	SM2	[26]
Bangladesh	223	1	1	45%	56%	73%	ALB	0.5	1/year	SM2	[27]
Oman	860	1	5	40%	0	0.5%	ALB	1–5	1/year	SM2	[28]
Ghana	1011	1	2	87%	NA	NA	ALB	1–2	2/year	SM2	[29]
Sri Lanka	177	1	5	84%	85%	72%	MEB	1–6	2/year	SM2	[30]
		26	45								

ALB, albendazole; MEB = mebendazole; IVR, ivermectin; DEC, diethylcarbamazine OM, original model; SM1, Simplified Model 1; SM2, Simplified Model 2

Programme coverage is an important parameter to consider when predicting changes in STH prevalence; however, coverage information was missing from the reports [16–30] and therefore when testing the models we assumed that the coverage of all the interventions exceeded 80% (i.e. the standard assumption in the model).

The time interval between collection of baseline data and follow-up data ranged between 6 months and 13 years (mean 3.2 years): in 24.4% of control programmes the interval between baseline and follow up was one year; in 37.5% the interval was between 2 and 4 years; in 35.6% the interval was 5–6 years; and in 2.3% the interval was more than 6 years.

At baseline the average prevalence of *A*. *lumbricoides* infection was 34% (range, 0–99%), of *T*. *trichiura* infection 27% (range, 0–100%) and of hookworm infection 45% (range, 0–99%).

At follow-up the observed average prevalence of *A*. *lumbricoides* infection was 13.9% (range, 0–75%), of *T*. *trichiura* infection 12.8% (range, 0–65%) and of hookworm infection 14% (range, 0–58%).

Some 53.3% of control programmes used albendazole, 24.3% used mebendazole and the remainder used a combination of drugs which were being used for the control of lymphatic filariasis (albendazole and either ivermectin or diethylcarbamazine).

We tested the different forms of the model (OM, SM1 and SM2) according to the kind of the data available from each programme:

- The performance of OM was tested in six follow-up datasets from Uganda and Viet Nam (i.e. Uganda, 2 year follow-up and 3 year follow-up; Viet Nam: 6 months follow-up, 2.5 year follow-up; 4.5 year follow-up; and 6 year follow-up). In both countries the data from the first year of follow-up after baseline were not used to evaluate the model because it had been used to define the TP matrix. Additionally, although the data from Viet Nam were already used to graphically test the first version of the model [4], in this evaluation we used different indicators (see below) and additional data (6th year after baseline).
- The performance of SM1 was tested on 18 follow-ups from eight countries.
- The performance of SM2 was tested on 45 follow-ups from 14 countries.

We developed two indicators to evaluate the accuracy of the model predictions for each dataset:

1. The discrepancy between predicted and observed data in absolute percentage points for each STH parasite infection. For example, if the prevalence predicted for hookworm infection was 5% and the observed prevalence was 10% we gave a value of 5 to this indicator. For each STH parasite we calculated the average discrepancy and the range.
2. The percentage of accurate predictions within 5 percentage points, between 5 and 10 percentage points, and more than 10 percentage points.

To our knowledge, no other models predicting STH prevalence changes are available, so we assumed that a model predicting more than 70% of the cases within 10 percentage points would yield satisfactory results.

### Ethics statement

The study was conducted on secondary data, and all data were recorded and analysed anonymously.

## Results

### Standard TPMS

Details of the 10 standard TPMS are listed in S3 Additional File; each TPMS contains three TP matrices (one matrix for each STH species).

### Estimation of condition states from total parasite prevalence

The nine multinomial curves and regression coefficients used to estimate the CS when the parasite prevalence is known (but not the classes of intensity) are published elsewhere [6]. The curves and coefficients allowed us to display graphically and estimate mathematically the prevalence of heavy, moderate and light intensity infections for each STH parasite, and therefore to run the model in cases where baseline information on CS was not available.

### Testing of the model

In general the mean discrepancy for any of the three STH parasites between the observed and the predicted prevalence was 3.16 percentage points (SD, 4.162) for OM, 4.8 percentage points (SD, 6.075) for SM1 and 8.4 percentage points (SD, 12.484) for SM2; these differences are marginally statistically different (*p* = 0.0301).

Table 5 summarizes the mean discrepancy between predicted and observed prevalence values for each parasite and for each of the models tested.

**Table 5:** Mean discrepancy and accuracy of the predictions obtained by the Markov original (OM) and simplified models (SM1 and SM2).

		Accuracy of the predictions		
	Mean discrepancies between predicted and observed prevalence in absolute percentage points	Proportion of predictions ≤ 5 percentage points from the observed values	Proportion of predictions > 5 and ≤ 10 percentage points from the observed values	Proportion of predictions > 10 percentage points from the observed values
OM				
*A*. *lumbricoides*	2.66 (range 0–7)	83.3%	16.7%	0%
*T*. *trichiura*	0.66 (range 0–3)	100%	0%	0%
hookworm	6.16 (range 0–14)	50%	33.3%	16.7%
SM1				
*A*. *lumbricoides*	5 (range 0–27)	66.7%	27.8%	5.6%
*T*. *trichiura*	3.22 (range 0–15)	83.3%	5.6%	11.1%
hookworm	6.41 (range 0–25)	58.8%	11.8%	29.4%
SM2				
*A*. *lumbricoides*	10.6 (range 0–60)	60.5%	9.8%	30.2%
*T*. *trichiura*	6.4 (range 0–41)	74.4%	2.3%	23.3%
hookworm	8.4 (range0–44)	46.7%	24.4%	28.9%

**Fig 2:**
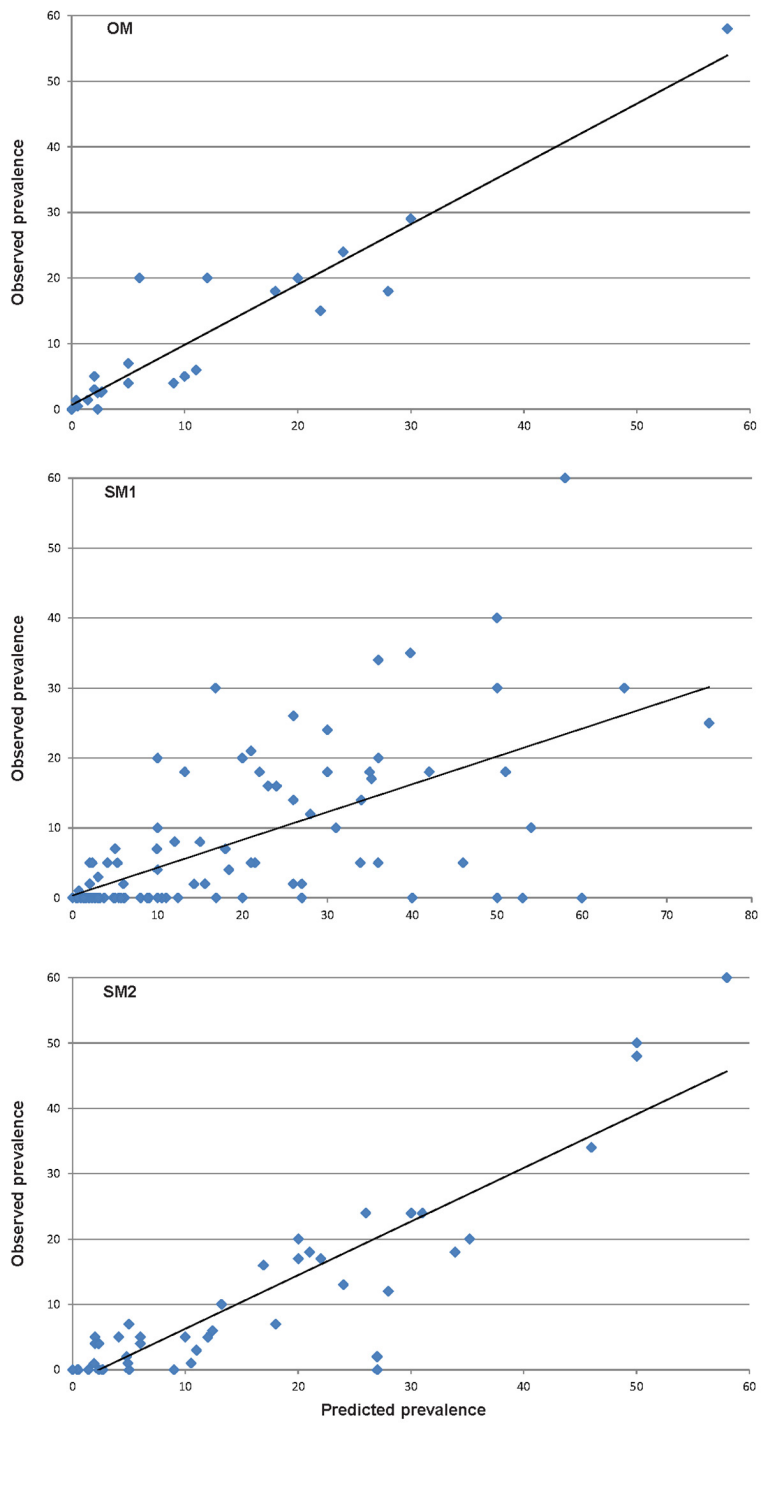
Relationship between observed and prevalence predicted by the original model (graph a) and simplified models (graphs a and b). Linear trend is included.

The accuracy of the models (proportion of predictions within 10 percentage points) for any of the STH parasites was 94% for OM, 84% for SM1 and 72% for SM2. In general the models preformed best for *T*. *trichiura* (43% of predictions within 1 percentage point) and least well for hookworm (only 26% of predictions within 1 percentage point). Table 5 presents details of the accuracy of the predictions by parasite and by model and Fig 2 illustrates for each dataset the observed prevalence and the prevalence predicted by OM, SM1 and SM2.

We also compared the precision of the predictions of OM, SM1 and SM2 only on the datasets from Viet Nam and Uganda, since these were the only datasets that were tested on all three models. Furthermore, this analysis (data not presented) showed a better performance of OM than SM1 and SM2 for all the three STH parasites; the differences are, however, not statistically significant.

We also evaluated the performance of the model using the Root Mean Square Error (RMSE) [32]. For OM the RMSE value was 0.04 (range of prediction discrepancy, 0–0.14), for SM1 the RMSE value was 0.11 (range of prediction discrepancy, 0–0.27) and for SM2 the RMSE value was 0.15 (range of prediction discrepancy 0–0.60), showing a precision progressively declining from OM1 to SM1 and SM2.

In the datasets where the intensity of infection was reported over time [5, 10], the model could predict with less than 5% discrepancy the prevalence of the different classes of intensity.

### Software available online

The software was designed to facilitate the application of the principle of the Markov model by managers of STH control programmes and is available online at: https://github.com/namkyodai/STHpredictor

The software features are described in the S4 Additional File.

## Discussion

Markov modelling has been used in trachoma and schistosomiasis control [33–34] but to our knowledge this is the first application for the prediction of changes in STH prevalence.

The Markov model for STH was developed with the aim of providing managers of control programmes with a simple tool to predict the STH prevalence (including the prevalence of each of the intensity categories by species) on a short- to medium-term basis (i.e. 1–5 years of implementation) and the simplified models (SM1 and SM2) were developed to allow a prediction with a more limited amount of data.

Since details on programme coverage were not available we assumed that it exceeded 80%. This lack of detailed information on coverage may have potentially reduced the precision of the model predictions.

The predicted prevalence values were less precise when the simplified models were used (OM performed better than SM1 and SM1 performed better than SM2). However, we recognize that frequently a complete set of individual baseline data and 1 year follow-up data (necessary to run OM) is not available to control programmes manager and therefore SM1 and SM2 could be the only option to obtain predictions on the changes to STH prevalence in real-world settings.

The largest discrepancies between predicted and observed prevalence were observed in surveys conducted in Sri Lanka and because no information on intensity of infection was available were analysed with SM3 only; the baseline prevalence for the three STH infections was extremely high (> 98%). It is likely that the method used for to estimate the class of intensity used by SM2 is not effective at very high prevalence, as the intensities of infection were likely much higher than those estimated for such datasets.

Generally, all the versions of the model (OM, SM1 and SM2) better predicted the prevalence changes for *A*. *lumbricoides* (predictions within 10 percentage points: OM, 100%; SM1, 94%; SM2, 70%) than for *T*. *trichiura* (predictions within 10 percentage points: OM, 100%; SM1, 88%; SM2, 75%) and hookworms (predictions within 10 percentage points: OM, 83%; SM1, 70%; SM2, 71%).

The model tested here assumes the “stationarity” of the TP and it is possible that without this assumption the predictions, especially the one at long term, could be further improved. We consider the prediction provided by the tool to be sufficiently precise to facilitate the planning and evaluation of programme performance, and concluded that the “stationarity” of the TP is an acceptable assumption for predicting STH changes in prevalence.

### Potential uses

The tool was developed mainly for use in STH control programmes by managers wishing to:

1. Evaluate the impact of preventive chemotherapy on the prevalence of STH infection: any large discrepancies between prevalence predicted by the model and that observed would require further investigation on the possible causes, including poor coverage, incomplete compliance, poor drug quality, inaccurate reporting and drug resistance. WHO has published a manual to assess the efficacy of drugs used in STH control programmes [32].
2. Programme managers can use the tool to compare the expected results of alternative interventions designs, for example by comparing the predicted prevalence after 5 years using mebendazole treatment versus albendazole, or administering treatment once per year versus twice per year. The model outputs could help programme managers decide on the more cost–effective intervention plan for their country.

It is important that programme managers understand the limitations of these models. Here, we do not aim to model explicitly the many dynamic elements that will affect the level of transmission, such as the rates of contamination of the environment, water contact, parasite acquisition, and any age-related and immune-related processes that may affect these rates. Instead, the modelling approach is to implicitly combine all of these elements into a composite measure of transmission.

Using the current approach, it is not possible to decipher which of the contributing factors leads to any lower than expected reductions in infection. Rather, where infection levels are observed to not being reduced as expected, this should prompt further investigation as to the underlying cause.

The model’s predictive capacity was lowered at the extreme upper end of the observed prevalence range. As mentioned above, the SM2 model appeared to predict better in case prevalence is less than 90%. In case of very high prevalence such as that observed in Sri Lanka in our dataset (> 98% prevalence), where it is likely that the proportion of individuals with high intensity of infection was underestimated for the CS. These are relatively rare values of infection in large-scale control programmes, and therefore fewer such data points are available to test the models.

All versions of the model predicted the expected prevalence of hookworms less precisely than for the other STH species. This is probably because hookworms are in reality two different species with different sensitivity to anthelminthics but were constantly reported collectively as “hookworms” in all the studies we considered.

For the purposes of medium- to short-term estimates (1–5 follow up years after baseline) we consider that models provide useful information for control programme managers. Further testing of the model against additional datasets would allow a better assessment of the predictive capacity of the model. We therefore invite researchers and managers of STH control programmes to download the β version of the software, to test it directly with their data and to report back to the authors.

We are now refining the software by linking it to a mapping tool to enable the development of maps illustrating the expected epidemiological changes as well as to estimate the costs and anthelminthics needed using population data and unit costs. Initial prototypes of this tool are being tested.

## Supporting Information

S1 Additional File Mathematical aspects. (DOCX)

S2 Additional File Values of coefficients used for simplified model 1 (SM1) and simplified model 2 (SM2). (DOCX)

S3 Additional File Standard transition probability matrix sets developed for use in the Markov model. (PDF)

S4 Additional File Software features. (DOCX)
