# Supplementary material for: Markov Model Predicts Changes in STH Prevalence during Control Activities Even with a Reduced Amount of Baseline Information
Source: PLoS Negl Trop Dis. 2016 Apr 1;10(4):e0004371. doi: 10.1371/journal.pntd.0004371 (PMC4817985; doi:10.1371/journal.pntd.0004371)
Supplement: S1 Additional File — (DOCX) [file pntd.0004371.s001.docx]

**S1 Additional file : Mathematical aspects**

Original Model (OM)

In mathematical terms, the baseline prevalence of an STH infection can be described by 4 condition states (CS), and the changes in the prevalence of infection occurring every year as a result of a pharmacological intervention can be represented by the transition probability (TP) through discrete condition states i ϵ I, I={1,2,3,4}, where 1,2,3 and 4 represent states of no infection, light, moderate and heavy intensity infections, respectively.

A symmetrical I x I matrix is produced for a time period of 1 year, called the TP matrix (P) and can be described in cardinal form as:

| $P=\left[\begin{matrix} p_{11} & p_{12} & \ldots& p_{1I} \\ p_{21} & p_{22} & \ldots& p_{I2} \\ \vdots& \vdots& \ddots& \vdots\\ p_{I1} & p_{I2} & \ldots& p_{II} \end{matrix}\right]$ | (1) |
| --- | --- |

Where p_ij_ represents the probability that an individual will transition from state i to state j after one cycle of preventative chemotherapy (one year after receiving the intervention).

The one year transition probability is estimated using following equation.

| 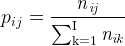 (2) |
| --- |

where

$nij$ is the number of individuals who transition from state i to state j in one cycle.

The prevalence of the STH intensity categories in the following year can be then calculated with the following equation:


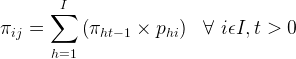
 (3)

where

t is the cycle number,

t-1 is the previous cycle,

t = 0 represents baseline prevalence,

π_it_ is the prevalence of state i at cycle number t *p hi* is the transition probability (i.e. the probability that an individual in state *h* transits to state *i)*.

Simplified Model (SM1)

In mathematical terms when only baseline data were available the TPs were estimated with the following equation
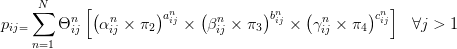
 (4)


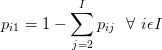


Where

$\theta_{ij}^{n}$, $\alpha_{ij}^{n}$, $\beta_{ij}^{n}$, $\gamma_{ij}^{n}$, $a_{ij}^{n}$, $b_{ij}^{n}$, and $c_{ij}^{n}$are coefficients determined by programme data or by extrapolation

$\pi_{2},\pi_{3},\pi_{4}$are the prevalence of infections of light intensity, medium intensity, and heavy intensity, respectively

n is the number of cycles of preventative chemotherapy

The values of these coefficients are given for each species and for each pair of possible transitions from i to j, in S2 Additional file.

Simplified Model 2 (SM2)

In mathematical terms, the intensity of each species was estimated using the following equation

| 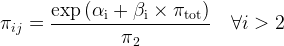 (5) |
| --- |


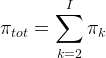


where α_i_ and β_i_ are regression coefficients of a model predicting prevalence of different classes of intensity published elsewhere (6),

*πtot* is the total prevalence of STH

For each STH parasite, these equations estimate the prevalence of light (CS2), moderate (CS3) and high (CS4) intensity, from the total prevalence at baseline (1-CS1).
