# Supplementary material for: Markov Model Predicts Changes in STH Prevalence during Control Activities Even with a Reduced Amount of Baseline Information
Source: PLoS Negl Trop Dis. 2016 Apr 1;10(4):e0004371. doi: 10.1371/journal.pntd.0004371 (PMC4817985; doi:10.1371/journal.pntd.0004371)
Supplement: S2 Additional File — (DOCX) [file pntd.0004371.s002.docx]

# S2 Additional file:

# Coefficients values used for Simplified Model 1 and 2

# Simplified Model 1 (SM1)

Note that the coefficients $\theta_{ij}^{n}$=0 for any transition to CS 4 and for any transition that is not shown in following tables.

Table A.1 : Coefficient values - Hookworm (TPMS 1)

| Total number of sub equations | Transition from | Values of coefficients | | | | | | | |
| --- | --- | --- | --- | --- | --- | --- | --- | --- | --- |
| N=4 | $i\to j$ | $\theta_{ij}^{n}$ | | $\alpha_{ij}^{n}$ | $\beta_{ij}^{n}$ | $\gamma_{ij}^{n}$ | $a_{ij}^{n}$ | $b_{ij}^{n}$ | $c_{ij}^{n}$ |
|  |  | VTN | ZNZ |  |  |  |  |  |  |
| n=1 | 1🡪2 | 1 | 4 | NA | 1 | 1 | 0 | 1 | 1 |
|  | 1🡪3 | 0.25 | 0.35 | NA | 1 | 1 | 0 | 1 | 1 |
|  | 2🡪2 | 0.4 | 0.8 | 1 | 1 | 1 | 1 | 1 | 1 |
|  | 2🡪3 | 0.1 | 0.4 | NA | 1 | 1 | 0 | 1 | 1 |
|  | 3🡪2 | 1.1 | 1.5 | NA | 3 | NA | 0 | 1 | 0 |
|  | 3🡪3 | 0.2 | 0.6 | NA | 1 | NA | 0 | 1 | 0 |
| n=2 | 1🡪2 | 1 | 4 | NA | 1 | NA | 0 | 1 | 0 |
|  | 1🡪3 | 0.25 | 0.35 | NA | 1 | NA | 0 | 1 | 0 |
|  | 2🡪2 | 0.4 | 0.8 | 1 | 1 | NA | 1 | 1 | 0 |
|  | 2🡪3 | 0.1 | 0.4 | NA | 1 | NA | 0 | 1 | 0 |
|  | 3🡪2 | 1.1 | 1.5 | NA | NA | 1 | 0 | 0 | 1 |
|  | 3🡪3 | 0.2 | 0.6 | NA | NA | 2 | 0 | 0 | 1 |
| n=3 | 2🡪2 | 0.4 | 0.8 | 1 | NA | NA | 1 | 0 | 0 |
| n=4 | 2🡪2 | 0.4 | 0.8 | NA | 1 | NA | 0 | 1 | 0 |

Table A.2 : Coefficient values - *A. lumbricoides* (TPMS 1)

| Total number of sub equations | Transition from | Values of coefficients | | | | | | | |
| --- | --- | --- | --- | --- | --- | --- | --- | --- | --- |
| N=2 | $i\to j$ | $\theta_{ij}^{n}$ | | $\alpha_{ij}^{n}$ | $\beta_{ij}^{n}$ | $\gamma_{ij}^{n}$ | $a_{ij}^{n}$ | $b_{ij}^{n}$ | $c_{ij}^{n}$ |
|  |  | ALB | MEB |  |  |  |  |  |  |
| n=1 | 1🡪2 | 0.3 | 1 | 1 | 2 | 3 | 1 | 1 | 1 |
|  | 1🡪3 | 0.4 | 0.7 | NA | 2 | 3 | 0 | 1 | 1 |
|  | 2🡪2 | 1 | 2 | 1 | 2 | 3 | 1 | 1 | 1 |
|  | 2🡪3 | 1 | 1 | NA | 2 | 3 | 0 | 1 | 1 |
|  | 3🡪2 | 1 | 1.4 | NA | 2 | 3 | 0 | 1 | 1 |
|  | 3🡪3 | 0.5 | 0.5 | NA | NA | 3 | 0 | 0 | 1 |
| n=2 | 1🡪2 | 0.3 | 0.4 | 1 | 2 | NA | 1 | 1 | 0 |
|  | 2🡪2 | 1 | 2 | 1 | 2 | NA | 1 | 1 | 0 |
|  | 3🡪2 | 1 | 1.4 | 1 | 2 | NA | 1 | 1 | 0 |

Table A.3 : Coefficient values - *T. trichiura* (TPMS 1)

| Total number of sub equations | Transition from | Values of coefficients | | | | | | | |
| --- | --- | --- | --- | --- | --- | --- | --- | --- | --- |
| N=4 | $i\to j$ | $\theta_{ij}^{n}$ | | $\alpha_{ij}^{n}$ | $\beta_{ij}^{n}$ | $\gamma_{ij}^{n}$ | $a_{ij}^{n}$ | $b_{ij}^{n}$ | $c_{ij}^{n}$ |
|  |  | VTN | ZNZ |  |  |  |  |  |  |
| n=1 | 1🡪2 | 0.25 | 0.5 | 1 | 1 | 1 | 1 | 1 | 1 |
|  | 1🡪3 | 0.2 | 0.3 | NA | 1 | 1 | 0 | 1 | 1 |
|  | 2🡪2 | 0.35 | 0.6 | 1 | 1 | 1 | 1 | 1 | 1 |
|  | 2🡪3 | 0.2 | 0.3 | NA | 1 | 1 | 0 | 1 | 1 |
|  | 3🡪2 | 1 | 0.8 | NA | 3 | NA | 0 | 1 | 0 |
|  | 3🡪3 | 0.125 | 0.1 | NA | 1 | NA | 0 | 1 | 0 |
| n=2 | 1🡪2 | 0.25 | 0.5 | 1 | 1 | NA | 1 | 1 | 0 |
|  | 2🡪2 | 0.35 | 0.6 | 1 | 1 | NA | 1 | 1 | 0 |
|  | 3🡪3 | 0.125 | 0.1 | NA | NA | 2 | 0 | 0 | 1 |
| n=3 | 1🡪2 | 0.25 | 0.5 | 1 | NA | NA | 1 | 0 | 0 |
|  | 2🡪2 | 0.35 | 0.6 | 1 | NA | NA | 1 | 0 | 0 |
| n=4 | 1🡪2 | 0.25 | 0.5 | NA | 1 | NA | 0 | 1 | 0 |
|  | 2🡪2 | 0.35 | 0.6 | NA | 1 | NA | 0 | 1 | 0 |

The values of coefficients$\alpha_{ij}^{n}$,$\beta_{ij}^{n}$,$\gamma_{ij}^{n}$,$a_{ij}^{n}$,$b_{ij}^{n}$,$c_{ij}^{n}$ are the same for all matrices. The only difference among them are the value of coefficient $\theta_{ij}^{n}$.

Table A.4 : Coefficient values - Hookworm (TPMS 2- TPMS 10)

| Total number of sub equations | Transition from | $\theta_{ij}^{n}$ | | | | | | | | | |
| --- | --- | --- | --- | --- | --- | --- | --- | --- | --- | --- | --- |
| N=4 | $i\to j$ | TPMS 2 | | TPMS 3 TPMS 9 TPMS 10 | | TPMS 4 | | TPMS 5 | | TPMS 6 TPMS 7 TPMS 8 | |
|  |  | VTN | ZNZ | VTN | ZNZ | VTN | ZNZ | VTN | ZNZ | VTN | ZNZ |
| n=1 | 1🡪2 | 1 | 4 | 0.15 | 4 | 0.2 | 4 | 0.9 | 4 | 1 | 4 |
|  | 1🡪3 | 0.35 | 0.35 | 0.25 | 0.35 | 0.25 | 0.35 | 0.2 | 0.35 | 0.2 | 0.35 |
|  | 2🡪2 | 0.5 | 0.8 | 0.4 | 0.8 | 0.4 | 0.8 | 0.4 | 0.8 | 0.4 | 0.8 |
|  | 2🡪3 | 0.2 | 0.4 | 0.1 | 0.8 | 0.1 | 0.4 | 0.1 | 0.4 | 0.1 | 0.4 |
|  | 3🡪2 | 1.5 | 1.5 | 1.1 | 1.5 | 1.1 | 1.5 | 1 | 1.5 | 1.1 | 1.5 |
|  | 3🡪3 | 0.3 | 0.6 | 0.2 | 0.6 | 0.2 | 0.6 | 0.2 | 0.6 | 0.2 | 0.6 |
| n=2 | 1🡪2 | 1 | 4 | 0.15 | 4 | 0.2 | 4 | 0.9 | 4 | 1 | 4 |
|  | 1🡪3 | 0.35 | 0.35 | 0.25 | 0.35 | 0.25 | 0.35 | 0.25 | 0.35 | 0.2 | 0.35 |
|  | 2🡪2 | 0.5 | 0.8 | 0.4 | 0.8 | 0.4 | 0.8 | 0.4 | 0.8 | 0.4 | 0.8 |
|  | 2🡪3 | 0.2 | 0.4 | 0.1 | 0.8 | 0.1 | 0.4 | 0.1 | 0.4 | 0.1 | 0.4 |
|  | 3🡪2 | 1.5 | 1.5 | 1.1 | 1.5 | 1.1 | 1.5 | 1.1 | 1.5 | 1.1 | 1.5 |
|  | 3🡪3 | 0.3 | 0.6 | 0.2 | 0.6 | 0.2 | 0.6 | 0.2 | 0.6 | 0.2 | 0.6 |
| n=3 | 2🡪2 | 0.5 | 0.8 | 0.4 | 0.8 | 0.4 | 0.8 | 0.4 | 0.8 | 0.4 | 0.8 |
| n=4 | 2🡪2 | 0.5 | 0.8 | 0.4 | 0.8 | 0.4 | 0.8 | 0.4 | 0.8 | 0.4 | 0.8 |

Table A.5 : Coefficient values - *A. lumbricoides* (TPMS 2- TPMS 10)

| Total number of sub equations | Transition from | $\theta_{ij}^{n}$ | | | | | | | | | |
| --- | --- | --- | --- | --- | --- | --- | --- | --- | --- | --- | --- |
| N=2 | $i\to j$ | TPMS 2 | | TPMS 3- TPMS 4 | | TPMS 5- TPMS 6- TPMS 7- TPMS 8 | | TPMS 9 | | TPMS 10 | |
|  |  | ALB | MEB | ALB | MEB | ALB | MEB | ALB | MEB | ALB | MEB |
| n=1 | 1🡪2 | 0.3 | 1 | 0.1 | 1 | 0.2 | 1 | 0.09 | 1 | 0.09 | 1 |
|  | 1🡪3 | 0.4 | 0.7 | 0.4 | 0.7 | 0.3 | 0.7 | 0.4 | 0.7 | 0.4 | 0.7 |
|  | 2🡪2 | 1 | 2 | 1 | 2 | 1 | 2 | 1 | 2 | 1 | 2 |
|  | 2🡪3 | 1 | 1 | 1 | 1 | 1 | 1 | 1 | 1 | 1 | 1 |
|  | 3🡪2 | 1 | 1.4 | 1 | 1.4 | 1 | 1.4 | 1 | 1.4 | 1 | 1.4 |
|  | 3🡪3 | 0.5 | 0.5 | 0.5 | 0.5 | 0.5 | 0.5 | 0.5 | 0.5 | 0.5 | 0.5 |
| n=2 | 1🡪2 | 0.3 | 1 | 0.1 | 1 | 0.2 | 1 | 0.2 | 1 | 0.2 | 1 |
|  | 2🡪2 | 1 | 2 | 1 | 2 | 1 | 2 | 1 | 2 | 1 | 2 |
|  | 3🡪2 | 1 | 1.4 | 1 | 1.4 | 1 | 1.4 | 1 | 1.4 | 1 | 1.4 |

Table A.6 : Coefficient values - - *T. trichiura* (TPMS 2- TPMS 6)

| Total number of sub equations | Transition from | $\theta_{ij}^{n}$ | | | | | | | | | |
| --- | --- | --- | --- | --- | --- | --- | --- | --- | --- | --- | --- |
| N=4 | $i\to j$ | TPMS 2 | | TPMS 3 | | TPMS 4 | | TPMS 5 | | TPMS 6- TPMS 7 | |
|  |  | VTN | ZNZ | VTN | ZNZ | VTN | ZNZ | VTN | ZNZ | VTN | ZNZ |
| n=1 | 1🡪2 | 0.15 | 0.5 | 0.18 | 0.5 | 0.15 | 0.5 | 0.1 | 0.5 | 0.09 | 0.5 |
|  | 1🡪3 | 0.3 | 0.3 | 0.2 | 0.3 | 0.2 | 0.3 | 0.2 | 0.3 | 0.2 | 0.3 |
|  | 2🡪2 | 0.35 | 0.6 | 0.35 | 0.6 | 0.35 | 0.6 | 0.35 | 0.6 | 0.35 | 0.6 |
|  | 2🡪3 | 0.2 | 0.3 | 0.2 | 0.3 | 0.2 | 0.3 | 0.2 | 0.3 | 0.2 | 0.3 |
|  | 3🡪2 | 0.8 | 0.8 | 0.8 | 0.8 | 0.8 | 0.8 | 0.8 | 0.8 | 0.8 | 0.8 |
|  | 3🡪3 | 0.1 | 0.1 | 0.125 | 0.1 | 0.125 | 0.1 | 0.125 | 0.1 | 0.1 | 0.1 |
| n=2 | 1🡪2 | 0.15 | 0.5 | 0.18 | 0.5 | 0.15 | 0.5 | 0.1 | 0.5 | 0.09 | 0.5 |
|  | 2🡪2 | 0.35 | 0.6 | 0.35 | 0.6 | 0.35 | 0.6 | 0.35 | 0.6 | 0.35 | 0.6 |
|  | 3🡪3 | 0.1 | 0.1 | 0.125 | 0.1 | 0.125 | 0.1 | 0.125 | 0.1 | 0.1 | 0.1 |
| n=3 | 1🡪2 | 0.15 | 0.5 | 0.18 | 0.5 | 0.15 | 0.5 | 0.1 | 0.5 | 0.09 | 0.5 |
|  | 2🡪2 | 0.35 | 0.6 | 0.35 | 0.6 | 0.35 | 0.6 | 0.35 | 0.6 | 0.35 | 0.6 |
| n=4 | 1🡪2 | 0.15 | 0.5 | 0.18 | 0.5 | 0.15 | 0.5 | 0.1 | 0.5 | 0.09 | 0.5 |
|  | 2🡪2 | 0.35 | 0.6 | 0.35 | 0.6 | 0.35 | 0.6 | 0.35 | 0.6 | 0.35 | 0.6 |

Table A.6 : Coefficient values - *T. trichiura* (TPMS 8- TPMS 10)

| Total number of sub equations | Transition from | $\theta_{ij}^{n}$ | | | | | |
| --- | --- | --- | --- | --- | --- | --- | --- |
| N=4 | $i\to j$ | TPMS 8 | | TPMS 9 | | TPMS 10 | |
|  |  | VTN | ZNZ | VTN | ZNZ | VTN | ZNZ |
| n=1 | 1🡪2 | 0.1 | 0.5 | 0.14 | 0.5 | 0.12 | 0.5 |
|  | 1🡪3 | 0.2 | 0.3 | 0.2 | 0.3 | 0.2 | 0.3 |
|  | 2🡪2 | 0.35 | 0.6 | 0.35 | 0.6 | 0.35 | 0.6 |
|  | 2🡪3 | 0.2 | 0.3 | 0.2 | 0.3 | 0.2 | 0.3 |
|  | 3🡪2 | 0.8 | 0.8 | 0.8 | 0.8 | 0.8 | 0.8 |
|  | 3🡪3 | 0.1 | 0.1 | 0.125 | 0.1 | 0.125 | 0.1 |
| n=2 | 1🡪2 | 0.1 | 0.5 | 0.14 | 0.5 | 0.12 | 0.5 |
|  | 2🡪2 | 0.35 | 0.6 | 0.35 | 0.6 | 0.35 | 0.6 |
|  | 3🡪3 | 0.1 | 0.1 | 0.125 | 0.1 | 0.125 | 0.1 |
| n=3 | 1🡪2 | 0.1 | 0.5 | 0.14 | 0.5 | 0.12 | 0.5 |
|  | 2🡪2 | 0.35 | 0.6 | 0.35 | 0.6 | 0.35 | 0.6 |
| n=4 | 1🡪2 | 0.1 | 0.5 | 0.14 | 0.5 | 0.12 | 0.5 |
|  | 2🡪2 | 0.35 | 0.6 | 0.35 | 0.6 | 0.35 | 0.6 |

# Simplified Model 2 (SM2)

| Species | intensity | $\lambda_{i}$ | $\delta_{i}$ |
| --- | --- | --- | --- |
| Hookworm | i=3 | 0.4997490 | -2.8858900 |
|  | i=4 | 0.7000660 | -3.9671500 |
| *A. lumbricoides* | i=3 | 0.5005658 | -0.5078976 |
|  | i=4 | 0.7736967 | -2.8866398 |
| *T. trichiura* | i=3 | 0.4716740 | -1.5894070 |
|  | i=4 | 0.4993203 | -5.3941940 |
