# Supplementary material for: Markov Model Predicts Changes in STH Prevalence during Control Activities Even with a Reduced Amount of Baseline Information
Source: PLoS Negl Trop Dis. 2016 Apr 1;10(4):e0004371. doi: 10.1371/journal.pntd.0004371 (PMC4817985; doi:10.1371/journal.pntd.0004371)
Supplement: S3 Additional File — (PDF) [file pntd.0004371.s003.pdf]

S3 Additional file

Standard Trasition Probability Matrix Sets developed for use in Markov Model

Each TPMS correspond to a different intervention

Each TPMS is presented in a different page of this document

Each TPMS contains three TP matrices (one matrix for each STH species).

Summary

	1st round	2nd round
**TPMS 1**	albendazole	albendazole
**TPMS 2**	mebendazole	mebendazole
**TPMS 3**	albendazole	No intervention
**TPMS 4**	mebendazole	No intervention
**TPMS 5**	Albendazole+ IVR	albendazole
**TPMS 6**	Albendazole+ IVR	mebendazole
**TPMS 7**	Albendazole+ DEC	albendazole
**TPMS 8**	Albendazole+ DEC	mebendazole
**TPMS 9**	Albendazole+ IVR	No intervention
**TPMS 10**	Albendazole+ DEC	No intervention

TPMS 1 1st round ALB 2nd round ALB

Ascaris lumbricoides

	CS1	CS2	CS3	CS4
CS1	0.8110	0.1170	0.0720	0.0000
CS2	0.4300	0.3900	0.1800	0.0000
CS3	0.3700	0.4800	0.1500	0.0000
CS4	1.0000	0.0000	0.0000	0.0000

Trichuris trichiura

	CS1	CS2	CS3	CS4
CS1	0.6271	0.2926	0.0803	0.0000
CS2	0.5101	0.4096	0.0803	0.0000
CS3	0.2544	1.2040	0.0504	0.0000
CS4	1.0000	0.0000	0.0000	0.0000

Hookworms

	CS1	CS2	CS3	CS4
CS1	0.7250	0.2200	0.0550	0.0000
CS2	0.6540	0.3240	0.0220	0.0000
CS3	0.1500	0.7700	0.0800	0.0000
CS4	1.0000	0.0000	0.0000	0.0000

**TPMS**	**2**	**1st round**	**MEB**	**2nd rounc**	**MEB**

Ascaris lumbricoides

	CS1	CS2	CS3	CS4
CS1	0.8110	0.1170	0.0720	0.0000
CS2	0.4300	0.3900	0.1800	0.0000
CS3	0.3700	0.4800	0.1500	0.0000
CS4	1.0000	0.0000	0.0000	0.0000
	CS1-CS2	CS1-CS3	CS2-CS2	CS3-CS2

Trichuris trichiura

	CS1	CS2	CS3	CS4
CS1	0.7040	0.1756	0.1204	0.0000
CS2	0.5101	0.4096	0.0803	0.0000
CS3	(0.0035)	0.9632	0.0403	0.0000
CS4	1.0000	0.0000	0.0000	0.0000

Hookworms	CS1	CS2	CS3	CS4
CS1	0.7030	0.2200	0.0770	0.0000
CS2	0.5510	0.4050	0.0440	0.0000
CS3	(0.1700)	1.0500	0.1200	0.0000
CS4	1.0000	0.0000	0.0000	0.0000

TPMS 3 1st round ALB 2nd round No intervention

Ascaris lumbricoides

	CS1	CS2	CS3	CS4
CS1	0.9530	0.0230	0.0240	0.0000
CS2	0.7100	0.2300	0.0600	0.0000
CS3	0.6650	0.2600	0.0750	0.0000
CS4	1.0000	0.0000	0.0000	0.0000

Trichuris trichiura

	CS1	CS2	CS3	CS4
CS1	0.8044	0.1476	0.0480	0.0000
CS2	0.6650	0.2870	0.0480	0.0000
CS3	0.2850	0.6400	0.0750	0.0000
CS4	1.0000	0.0000	0.0000	0.0000

Hookworms	CS1	CS2	CS3	CS4
CS1	0.9706	0.0110	0.0184	0.0000
CS2	0.8788	0.1139	0.0074	0.0000
CS3	0.6800	0.2860	0.0340	0.0000
CS4	1.0000	0.0000	0.0000	0.0000

TPMS 4 1st round MEB 2nd rounc No intervention

Ascaris lumbricoides

	CS1	CS2	CS3	CS4
CS1	0.9530	0.0230	0.0240	0.0000
CS2	0.7100	0.2300	0.0600	0.0000
CS3	0.6650	0.2600	0.0750	0.0000
CS4	1.0000	0.0000	0.0000	0.0000

Trichuris trichiura

	CS1	CS2	CS3	CS4
CS1	0.9535	0.0364	0.0101	0.0000
CS2	0.8989	0.0910	0.0101	0.0000
CS3	0.8633	0.1280	0.0088	0.0000
CS4	1.0000	0.0000	0.0000	0.0000

Hookworms	CS1	CS2	CS3	CS4
CS1	0.9010	0.0440	0.0550	0.0000
CS2	0.7028	0.2752	0.0220	0.0000
CS3	0.1500	0.7700	0.0800	0.0000
CS4	1.0000	0.0000	0.0000	0.0000

TPMS 5 1st round ALB+IVR 2nd round ALB

Ascaris lumbricoides

	CS1	CS2	CS3	CS4
CS1	0.8680	0.0780	0.0540	0.0000
CS2	0.4300	0.3900	0.1800	0.0000
CS3	0.3700	0.4800	0.1500	0.0000
CS4	1.0000	0.0000	0.0000	0.0000

Trichuris trichiura

	CS1	CS2	CS3	CS4
CS1	0.9640	0.0260	0.0100	0.0000
CS2	0.8990	0.0910	0.0100	0.0000
CS3	0.8740	0.1208	0.0052	0.0000
CS4	1.0000	0.0000	0.0000	0.0000

Hookworms	CS1	CS2	CS3	CS4
CS1	0.7580	0.1980	0.0440	0.0000
CS2	0.6540	0.3240	0.0220	0.0000
CS3	0.2200	0.7000	0.0800	0.0000
CS4	1.0000	0.0000	0.0000	0.0000

TPMS 6 1st round ALB+IVR 2nd round MEB

Ascaris lumbricoides

	CS1	CS2	CS3	CS4
CS1	0.8680	0.0780	0.0540	0.0000
CS2	0.4300	0.3900	0.1800	0.0000
CS3	0.3700	0.4800	0.1500	0.0000
CS4	1.0000	0.0000	0.0000	0.0000

Trichuris trichiura

	CS1	CS2	CS3	CS4
CS1	0.8831	0.0729	0.0440	0.0000
CS2	0.6725	0.2835	0.0440	0.0000
CS3	0.4000	0.5600	0.0400	0.0000
CS4	1.0000	0.0000	0.0000	0.0000

Hookworms	CS1	CS2	CS3	CS4
CS1	0.7360	0.2200	0.0440	0.0000
CS2	0.6540	0.3240	0.0220	0.0000
CS3	0.2200	0.7000	0.0800	0.0000
CS4	1.0000	0.0000	0.0000	0.0000

TPMS **7** **1st round ALB+DEC 2nd round ALB**

Ascaris lumbricoides

	CS1	CS2	CS3	CS4
CS1	0.8680	0.0780	0.0540	0.0000
CS2	0.4300	0.3900	0.1800	0.0000
CS3	0.3700	0.4800	0.1500	0.0000
CS4	1.0000	0.0000	0.0000	0.0000

Trichuris trichiura

	CS1	CS2	CS3	CS4
CS1	0.8831	0.0729	0.0440	0.0000
CS2	0.6725	0.2835	0.0440	0.0000
CS3	0.4000	0.5600	0.0400	0.0000
CS4	1.0000	0.0000	0.0000	0.0000

Hookworms	CS1	CS2	CS3	CS4
CS1	0.7360	0.2200	0.0440	0.0000
CS2	0.6540	0.3240	0.0220	0.0000
CS3	0.2200	0.7000	0.0800	0.0000
CS4	1.0000	0.0000	0.0000	0.0000

TPMS 8 1st round ALB+DEC 2nd round MEB

Ascaris lumbricoides

	CS1	CS2	CS3	CS4
CS1	0.8680	0.0780	0.0540	0.0000
CS2	0.4300	0.3900	0.1800	0.0000
CS3	0.3700	0.4800	0.1500	0.0000
CS4	1.0000	0.0000	0.0000	0.0000

Trichuris trichiura

	CS1	CS2	CS3	CS4
CS1	0.9640	0.0260	0.0100	0.0000
CS2	0.8990	0.0910	0.0100	0.0000
CS3	0.8740	0.1208	0.0052	0.0000
CS4	1.0000	0.0000	0.0000	0.0000

Hookworms	CS1	CS2	CS3	CS4
CS1	0.7360	0.2200	0.0440	0.0000
CS2	0.6540	0.3240	0.0220	0.0000
CS3	0.2200	0.7000	0.0800	0.0000
CS4	1.0000	0.0000	0.0000	0.0000

TPMS 9 1st round ALB+IVR 2nd round No Intervention

Ascaris lumbricoides

	CS1	CS2	CS3	CS4
CS1	0.8929	0.0351	0.0720	0.0000
CS2	0.4300	0.3900	0.1800	0.0000
CS3	0.3700	0.4800	0.1500	0.0000
CS4	1.0000	0.0000	0.0000	0.0000

Trichuris trichiura

	CS1	CS2	CS3	CS4
CS1	0.9535	0.0364	0.0101	0.0000
CS2	0.8989	0.0910	0.0101	0.0000
CS3	0.8633	0.1280	0.0088	0.0000
CS4	1.0000	0.0000	0.0000	0.0000

Hookworms	CS1	CS2	CS3	CS4
CS1	0.9706	0.0110	0.0184	0.0000
CS2	0.8788	0.1139	0.0074	0.0000
CS3	0.6800	0.2860	0.0340	0.0000
CS4	1.0000	0.0000	0.0000	0.0000

TPMS 10 1st round ALB+DEC 2nd round No intervention

Ascaris lumbricoides

	CS1	CS2	CS3	CS4
CS1	0.8968	0.0312	0.0720	0.0000
CS2	0.4300	0.3900	0.1800	0.0000
CS3	0.3700	0.4800	0.1500	0.0000
CS4	1.0000	0.0000	0.0000	0.0000

Trichuris trichiura

	CS1	CS2	CS3	CS4
CS1	0.8588	0.0972	0.0440	0.0000
CS2	0.6725	0.2835	0.0440	0.0000
CS3	0.3900	0.5600	0.0500	0.0000
CS4	1.0000	0.0000	0.0000	0.0000

Hookworms	CS1	CS2	CS3	CS4
CS1	0.9128	0.0327	0.0545	0.0000
CS2	0.6546	0.3236	0.0218	0.0000
CS3	0.1650	0.7590	0.0760	0.0000
CS4	1.0000	0.0000	0.0000	0.0000
