# Supplementary material for: Markov Model Predicts Changes in STH Prevalence during Control Activities Even with a Reduced Amount of Baseline Information
Source: PLoS Negl Trop Dis. 2016 Apr 1;10(4):e0004371. doi: 10.1371/journal.pntd.0004371 (PMC4817985; doi:10.1371/journal.pntd.0004371)

**S3 Additional file**

**Software features**

The β version of the software is available for testing by researchers at: <https://github.com/namkyodai/STHpredictor>

The software was developed as a MS Window^®^ application and, as mentioned, includes three different modules (one for each of the three levels of data availability mentioned before):

**Module 1**

This module of the software requires, as .csv files, information on infection intensity class at baseline and after one year of intervention of at least 200 individuals. The software automatically calculates CS and TP and predicts the annual epidemiological situation for up to10 years after baseline.

**Module 2**

This module of the software offers the possibility to select among

three types of programmes

- school-based programme only;
- school-based programme integrated with the control of lymphatic filariasis;
- control of lymphatic filariasis only;

Two drugs used

- albendazole
- mebendazole

Two frequencies of administration

- twice
- once a year.

The software also considers the possible integration with a lymphatic filariasis elimination programme providing, to the entire population, albendazole with ivermectin or albendazole with diethylcarbamazine.

In addition to the set of TP calculated for the use of albendazole alone in Vietnam^4^, nine additional sets of TP were developed, each set of TP was derived from programmes implementing different strategies.

Briefly, in this module, the software allows to select a set of TP corresponding to the characteristic of the programme implemented, then apply it to the CS at baseline (baseline data including classes of intensity for each STH infection would need to be entered into the module).

**Module 3:**

Similarly to module 2, this module of the software offers the possibility to select among different types of programmes, drug used and different frequencies of administration and apply the same sets of TP.

In this module the software requires only baseline total prevalence of each STH and estimates the prevalences of the different CS (based on curves specifically developed for this purpose)^5^ and apply the estimated TP to the estimated CS.

In the three modules, when the entire population is treated, the predicted prevalence was constantly higher than the observed ones, this discrepancy between the two curves was related to the baseline prevalence: if the baseline prevalence was high (> 80%) the discrepancy started after five years, for lower initial prevalences the discrepancy started after 3 or 4 years. We interpret this discrepancy as a progressive reduction of reinfection due to the reduction of the soil contamination resulting from the control intervention: for this reason, we adjusted the predicting curves by including a standard modification, dependient from the initial level of prevalence. This adjustment is not applied when only a part of the population is treated (as in case of school deworming programmes).

The Appearance of the WHO software is presented in the following figure

**Additional file Figure:** Appearance of the WHO software

Software introduction screen


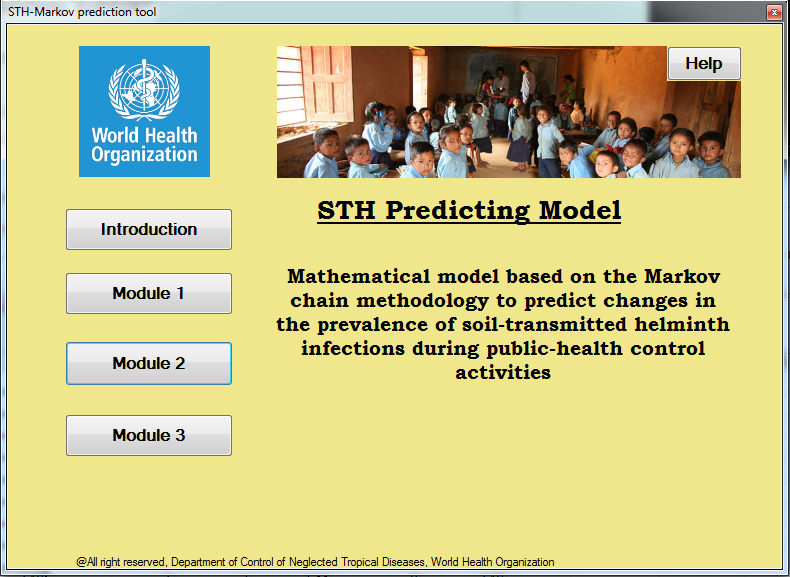


b) control programme information data entry


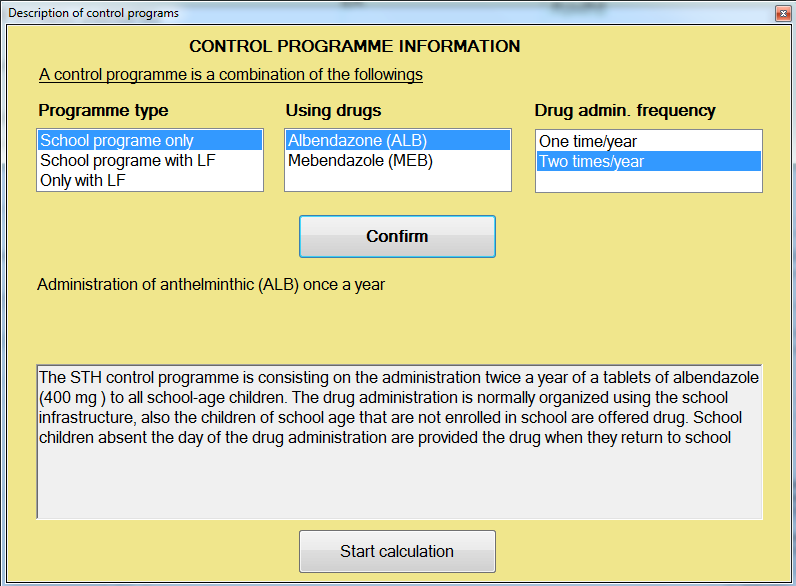


c) prevalence entry


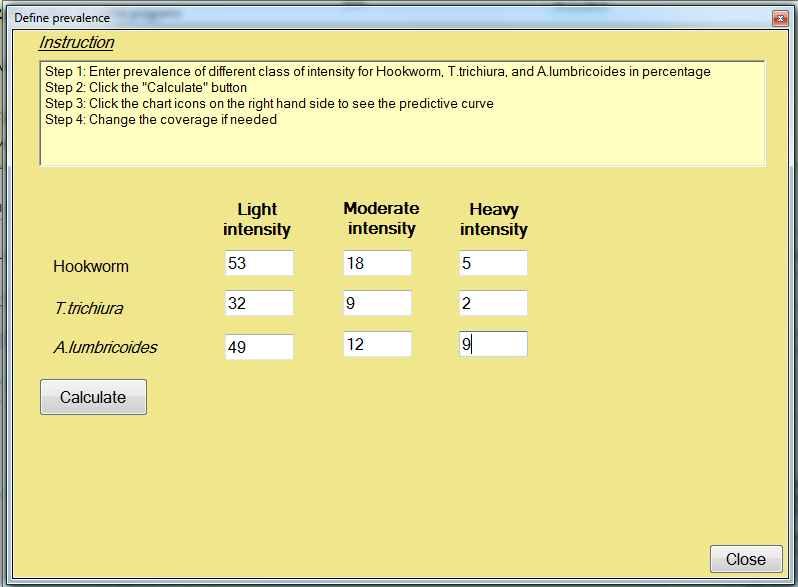


d) output: curves predicting changes in prevalence


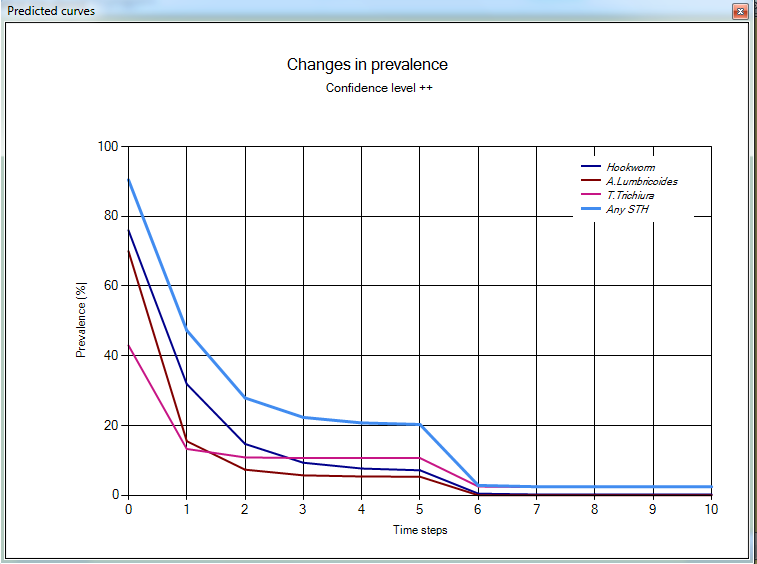

Supplement: S4 Additional File — (DOCX) [file pntd.0004371.s004.docx]
